# Versican and Tumor-Associated Macrophages Promotes Tumor Progression and Metastasis in Canine and Murine Models of Breast Carcinoma

**DOI:** 10.3389/fonc.2019.00577

**Published:** 2019-07-03

**Authors:** Diego Carlos dos Reis, Karine Araújo Damasceno, Cecília Bonolo de Campos, Emerson Soares Veloso, Gabriela Rafaela Arantes Pêgas, Lucas Rocha Kraemer, Michele Angela Rodrigues, Matheus Silvério Mattos, Dawidson Assis Gomes, Paula Peixoto Campos, Enio Ferreira, Remo Castro Russo, Geovanni Dantas Cassali

**Affiliations:** ^1^Department of General Pathology, Institute of Biological Sciences, Universidade Federal de Minas Gerais, Belo Horizonte, Brazil; ^2^Laboratory of Pulmonary Immunology and Mechanics, Department of Physiology and Biophysics, Institute of Biological Sciences, Universidade Federal de Minas Gerais, Belo Horizonte, Brazil; ^3^Oswaldo Cruz Foundation, Gonçalo Moniz Institute, Salvador, Brazil; ^4^Department of Biochemistry and Immunology, Institute of Biological Sciences, Universidade Federal de Minas Gerais, Belo Horizonte, Brazil

**Keywords:** breast cancer, angiogenesis, lung metastasis, versican, tumor-associated macrophages

## Abstract

Versican and tumor-associated macrophages (TAMs) are involved in growth and metastases in several cancers. Here, we investigated the potential role of versican, a matrix proteoglycan, and its correlation with TAMs infiltrates in different stages of two different breast cancer models: spontaneous canine mammary gland carcinomas and the murine 4T1 breast cancer model. The stromal versican expression was correlated with TAMs accumulation in tumors with an advanced stage from spontaneous canine mammary carcinoma samples. Versican expression in mice, identified in late stages of tumor progression, was associated to a high number of peri-tumoral infiltrating TAMs. Indeed, TAMs were related to a pro-inflammatory and pro-angiogenic state in the primary tumor. Furthermore, TAMs accumulation was related to versican expression in the lungs and an increased number of pulmonary metastatic nodules with pulmonary mechanical dysfunction, which was due to leukocyte influx in the airways and elevated growth factor levels in the microenvironment. Thus, we suggest that versican and TAMs as attractive targets for breast cancer therapy.

## Introduction

The tumor microenvironment has been increasingly recognized as an important participant of tumor progression (1, 2) and metastasis (3). As a result, there is an escalating interest in studies involving components of the extracellular matrix (ECM) and its interaction with neoplastic, stromal, endothelial and immune cells, as well fibroblasts (4–6). Interaction between ECM and inflammatory cells, mostly macrophages have been shown to be involved in the progression and development of breast cancer metastasis (7).

Breast cancer is the most common cancer in women worldwide and the second leading cause of cancer-related deaths in women (8). Generally, localized diseases are largely curable, whereas metastatic or recurrent diseases imply in poor prognosis. Metastatic disease may be found in broad range of organs and tissues, such as the bone, brain, liver and lungs (9). Lung metastasis may affect the pulmonary microenvironment and its function. Indeed, reduced lung function is an important risk factor for lung cancer, increasing the surgical risk of patients with advanced lung cancer, in which the low forced expiratory volume (FEV1) is strongly associated with mortality of non-small cell lung cancer patients (10).

Versican is an extracellular matrix proteoglycan that is highly expressed in early development stages, with low expression remaining in adults, increasing dramatically following tissue inflammation (11, 12) and neoplastic diseases (13). In cancer, versican has been identified as a modulator of cell adhesion, proliferation, apoptosis, angiogenesis, invasion, and metastasis (14–16). Recent studies also have been shown that versican interacts with monocytes/macrophages promoting their activation, migration, and production of growth factors. By activating TLR2:TLR6 complexes from tumor-associated macrophages (TAMs), versican induces the production of pro-inflammatory and pro-tumoral cytokines and chemokines, such as tumor necrosis factor-α (TNF-α), tumor growth factor-β1 (TGF-β1), vascular endothelial growth factor (VEGF), and CC chemokine ligand 2 (CCL2), as well as versican production (17–20).

Tumor-associated macrophages (TAMs) are found in different tumor types (21–23) and are related with poor prognosis (21, 24–26). TAMs closely resemble the M2-polarized macrophages, which promote tumor growth and progression by several mechanisms, including the secretion of pro-tumoral growth factors and inhibitory cytokines. In fact, TAMs also induce angiogenesis, reduce effector functions of tumor-infiltrating lymphocytes, and enhance T regulatory lymphocyte (Treg) expansion (27). The relationship between versican and TAMs in breast cancer development is still poorly understood. Therefore, the aim of this study was to characterize the expression of versican and TAMs in spontaneous primary canine mammary gland carcinomas and in the 4T1 mice model of breast cancer, further investigating their role during primary cancer progression and metastasis development in the context of breast cancer.

## Materials and Methods

### Canine Mammary Tumors Samples

In total, 108 cases of canine mammary tumors were selected from the archives of the Laboratory of Comparative Pathology of the Federal University of Minas Gerais (UFMG). The samples derived from female dogs of different breeds and ages, which had undergone surgical excision of the mammary gland neoplasm. The neoplasms were categorized according to the World Health Organization (WHO) classification scheme for canine mammary tumors (28) incorporating updated classification proposals (29). Histological grade was established according to the Nottingham system (30). All tumors were staged from I to V according to Owen (31). All procedures were performed under the guidelines and with the approval of the Ethics Committee in Animal Experimentation (CETEA/UFMG), protocol 219/2009 and 81/2013.

### 4T1 Mice Model of Breast Cancer

The 4T1 mouse mammary carcinoma was obtained from the American Type Culture Collection (Manassas, USA), and the 4T1 cells were maintained in RPMI 1640 medium supplemented with 10% fetal bovine serum (Hyclone, Logan, UT, USA). Cell cultures were maintained at 37°C in a humidified atmosphere of 5% CO_2._

Female BALB/c mice (16–20 g), with 6–8 week old (specific-pathogen-free) were obtained from the Centro de Bioterismo (CEBIO) of the Universidade Federal de Minas Gerais (UFMG), in Brazil. The mice were housed in a ventilated barrier rack (Alesco Indústria e Comércio Ltda, Monte Mor, SP, Brazil) in a temperature-controlled facility on a 12-h photoperiod. The mice were given food and water *ad libitum*. Mice were subcutaneously inoculated with 2.5 × 10^6^ 4T1 cells in the right flank. They were anesthetized with a subcutaneous injection of 8.5 mg/kg of xylazine and 130 mg/kg of ketamine on the 14th, 21st, and 28th day following tumor inoculation, to perform the assessment of respiratory mechanics and blood sample collection. Assessments of respiratory mechanics were also performed in control mice (*n* = 8). Then, under anesthesia, the tumor bearing-mice and control mice were euthanized and bronchoalveolar lavage (BAL) was performed. Tumor and lung samples were collected, weighted, and processed for biochemical and histological analysis. All research was conducted under a protocol approved (number 262/2012) by an Ethics Committee on Animal Use (CEUA) from the UFMG.

### Assessment of Respiratory Mechanics

While maintaining spontaneous breathing under anesthesia, the mice were tracheostomized, placed in a body plethysmograph and connected to a computer-controlled ventilator (Forced Pulmonary Maneuver System®, Buxco Research Systems, Wilmington, North Carolina USA) as previously described by Russo et al. (32). The parameters assessed were Forced Vital Capacity (FVC), Functional Residual Capacity (FRC), Residual Volume (RV), Pressure × Volume curve, Forced Expiratory Volume at 50 ms (FEV50), Forced Expiratory Volume at 100 ms (FEV100), Tiffeneau-Pinelli index (FEV50/FVC), Fast Flow Volume curve, Dynamic Compliance (Cdyn), and Lung Resistance (Rl).

### Bronchoalveolar Lavage (BAL) and Lung Preparation

BAL was performed to obtain leukocytes from the alveolar space. The number of leukocytes was determined by total counts in a Newbauer chamber, with differential counts performed in citospin preparations stained with May-Grunwald-GiemsaLi, as previously described (33). After BAL collection, 5 mL of saline-phosphate buffer (PBS) was administered in the right ventricle so that the pulmonary blood vessels and capillaries were washed. Afterwards, the left lung (single lobe) was collected for histological analysis and the right lung was collected, weighed, and frozen for further processing and analysis of cytokines, chemokines, and N-Acetyl-β-glucosaminidase (NAG).

### Histopathological Analysis

Primary tumors and lungs were excised and fixed in 10% neutral buffered formalin (pH 7.4) for 48 h and embedded in paraffin. One 4 μm-thick sections were obtained and stained with hematoxylin and eosin (H&E) and examined under light microscopy by two pathologists blinded to the experiment. Neoplastic cellular nodules or aggregates distributed throughout the lung parenchyma or alveolar spaces were considered metastatic lesions. Vascular density was performed on primary tumor slides stained with Gomori's trichrome in five fields (20x objective), as previously described (33, 34). Collagen deposition was analyzed with the WCIF ImageJ software (NHI) (http://www.uhnresearch.ca/facilities/wcif/imagej/).

### Immunohistochemistry

Versican immunolabeling was performed in canine and murine tumor sections subjected to heat-induced antigen retrieval with chondroitinase ABC (Proteus vulgaris; Sigma Chemicals) digestion at 37°C for 90 min with 0.5 U/mL of the enzyme in 0.25 M Tris buffer (pH 8.0) containing 0.18 M sodium chloride and 0.05% bovine serum albumin (BSA). Next, 0.25 M Tris buffer (pH 8.0) containing 0.1 M 6-amino-n-caproic-acid and 5 mM benzamidine hydrochloride was added and the samples were incubated for 30 min to inhibit protease activity. Murine macrophage immunolabeling was performed in tumor sections subjected to antigen retrieval with pepsin (Merck, Billerica, MA, USA) (100 mg pepsin dissolved in 100 ml H_2_O and 1 ml HCl 1N) for 30 min at 37°C. Endogenous peroxidase activity was blocked with 3% hydrogen peroxidase in methanol. Slides were then washed and incubated overnight at 4°C with 12C5 (Cambridge, UK) for versican and F4/80 (BM8, Hycult Biotech, Netherlands) for macrophages, both monoclonal antibodies. A polymer detection system was used for the identification of the secondary antibody (ADVANCE HRP-ready to use, DakoCytomation). Diaminobenzidine (DAB) was used as a chromogen and sections were counterstained with Mayer's hematoxylin. Negative controls were obtained by substitution of primary antibody by normal serum. Tissue obtained from a newborn mouse brain and the spleen of Balb/c mice were used as positive controls for versican and macrophages, respectively. Canine macrophages immunolabeling was performed as previously described (34).

Murine macrophages stained by F4/80 were counted by selecting 10 hotspot fields at high magnification (400X). Canine macrophages stained by MAC387 were counted in 5 hotspot fields. Versican expression was evaluated in stromal areas adjacent to the neoplasm semi-quantitatively based on the system of score adapted from Skandalis et al. (35), through the overall percentage of the tissue section stained positive (0–100%) and the 4-point signal intensity scale, classified as: 1, negative or very weak staining; 2, weak staining; 3, moderate staining; and 4, strong staining. Versican expression in neoplastic cells was evaluated considering the same score of intensity and percentage only for the 4T1 tumor model.

### Tissue Extraction and Determination of N-acetyl-β-D-glucosaminidase Activity and Vascularization

The infiltration of macrophages was quantified by measuring the levels of the lysosomal enzyme N-acetyl-β-D-glucosaminidase (NAG) present in the lungs, as previously described (36). One hundred milligrams of primary tumors and the right lung were homogenized and centrifuged with 100 mL of the supernatant was collected and incubated for 10 min with p-nitrophenyl-N-acetyl-β-D-glucosaminide (Sigma). The reaction was stopped with the addition of 0.2 M glycine buffer (pH 10.6). Results were expressed as nmol/mg of wet tissue. The vascularization extent in tumor samples was assessed by the amount of hemoglobin (Hb) detected in the tissue using the Drabkin method, as described by Ferreira et al. (37).

### Immunofluorescence

Confocal immunofluorescence was performed as previously described, with minor changes (38). Primary antibodies used for immunofluorescence were FITC-conjugated anti-F4/80 (1:150, clone mAb BM8, FITC conjugated, Hycult Biotech, Uden, The Netherlands) and anti-TGF-β1 (1:100, polyclonal IgG, Santa Cruz Biotechnology, USA), with Alexa Fluor 647 (1:1,000, Life Technologies) as the secondary antibody. Nuclei were stained with Hoechst dye 33258 (1 μg/mL, Life Technologies) before coverslip mounting for immunofluorescence and confocal laser scanning microscopy (Zeiss LSM 5 Live, Carl Zeiss, Jena, Germany) (40x).

### Enzyme-Linked Immunosorbent Assay (ELISA)

Primary tumor and right lung samples were homogenized and centrifuged at 4°C for 10 min at 10,000 g. Then, TNF-α, TGF-β1, VEGF, and CCL2 levels were quantified in primary tumor supernatants and lung homogenates were quantified for CXCL1, CCL2, VEGF, and TGF-β1 using the DuoSet ELISA kits (R&D Systems) in accordance to the manufacturer's instructions, as previously described (32). The results were expressed in picograms per milligram of wet tissue.

### Statistical Analysis

Data are presented as the mean ± SEM and analyzed by one-way ANOVA, with the differences between groups assessed using the Student-Newman-Keuls *post-hoc* test. Differences in versican expression in the different stages of tumor progression were evaluated by the Kruskal–Wallis test, with the differences between groups assessed using the Mann–Whitney *post-hoc* test. Correlations were analyzed by the Spearman test. Graphs and analysis were performed using the GraphPad Prism 5.0 software. Differences were considered statistically significant when *p* < 0.05.

## Results

### Stromal Versican Expression Correlates With TAM Accumulation in Low-Grade and Advanced Stages of Canine Mammary Carcinomas

**Figure 1:**
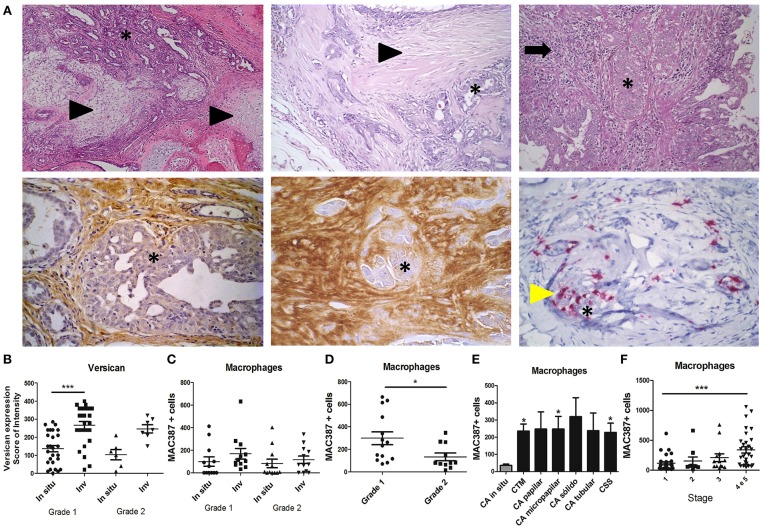
Expression and distribution of versican and TAMs in spontaneous canine mammary carcinomas. Carcinoma in mixed tumors **(A)** presents epithelial (asterisk) and mesenchymal components (arrowhead) (Upper panel, left and middle images) often surrounded by inflammatory and stromal cells (arrow, upper panel, right image). Immunohistochemistry analysis shows versican expression in the stroma of *in situ* areas of carcinoma in mixed tumors (bottom panel, left image) and invasive stroma areas (middle image). MAC387+ macrophages (yellow arrowhead, bottom panel, right image) are shown infiltrating around epithelial neoplastic areas of carcinoma in mixed tumors. Versican expression in carcinomas in mixed tumors was increased in invasive areas when compared to *in situ* areas of grade 1 tumors and *in situ* areas of grade 2 tumors **(B)**. MAC387+ macrophages quantified by immunohistochemistry were not significantly different across *in situ* and invasive areas of carcinomas in mixed tumors **(C)**. Grade I carcinoma in mixed tumor presented higher infiltration of MAC387+ macrophages when compared to grade 2 **(D)**. Diverse invasive carcinomas presented higher infiltration of MAC387+ macrophages when compared to *in situ* carcinomas **(E)**. Stage 4 and 5 tumors presented higher infiltration of MAC387+ macrophages when compared to stage 1 **(F)**. Results are shown as the mean ± SEM. Kruskal–Wallis test followed by Mann–Whitney test was used to evaluate differences in **(C)**. ^*^P < 0.05, ^***^P < 0.001, respectively.

We first examined the expression of versican and TAMs infiltration in 108 spontaneous canine mammary neoplasms. Carcinomas in mixed tumor, the most common histological type diagnosed in neoplasm of the mammary gland of dogs (30), were used to evaluate versican expression in *in situ* and invasive areas (Figure 1A). Versican expression was observed in the stroma, myxoid matrix, and endothelial cells of blood vessels, inflammatory cells, and fibroblasts. Carcinomas in mixed tumors classified as histological grade 1 presented higher versican expression in invasive areas when compared to *in situ* areas (*P* < 0.001) (Figure 1B). TAMs were evaluated in *in situ* and invasive areas of diverse histological types (Figure 1C). Grade 1 tumors presented increased TAMs than grade 2 tumors (Figure 1D). TAM was increased in invasive carcinomas throughout all histological types, with statistical significance in carcinomas in mixed tumors, micropapillary carcinomas, tubular carcinomas, and carcinosarcomas, when compared to *in situ* carcinomas (Figure 1E). When we categorized these tumors by clinical stage, TAMs infiltration was increased in tumors with advanced clinical stage when compared to early stage tumors (Figure 1F).

### Stromal Expression of Versican Is Related With Peritumoral TAM Infiltration and Tumor Progression in Primary Mammary Tumors Using the 4T1 Murine Breast Cancer Model

**Figure 2:**
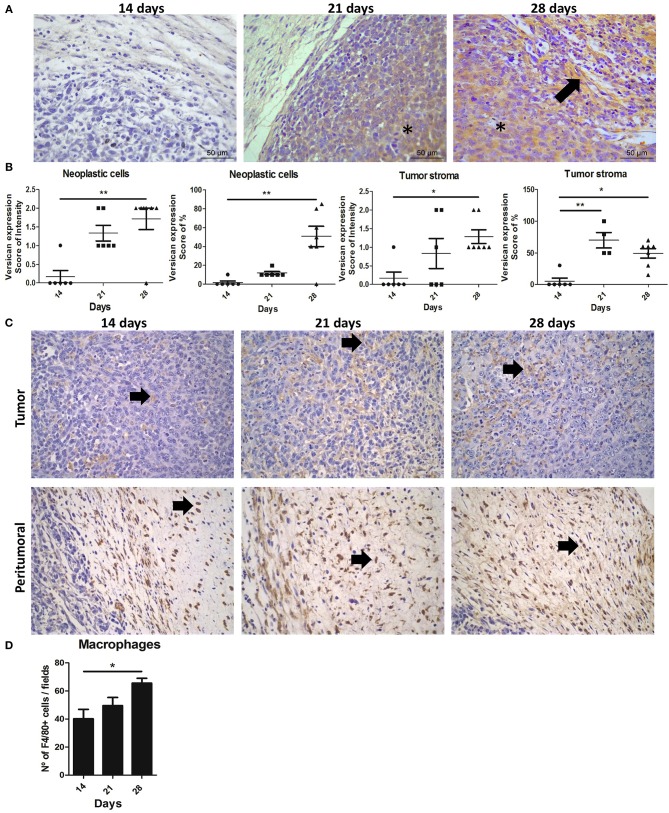
Expression and distribution of versican and TAMs during 4T1 mammary carcinoma progression in mice. Balb/c mice were inoculated with 2.5 × 10^6^ 4T1 cells in the right flank area and follow up to the 14th, 21st, and 28th days of tumor-transplant. Immunohistochemistry shows tumors on 28th day exhibiting a strong and diffuse versican expression in peritumoral areas and neoplastic cells when compared to tumors on 14th day post-transplant **(A)**, and quantification on **(B)**. Arrows indicate positive staining (brown) in peritumoral areas of the tumor. Asterisks indicate positive staining (brown) in neoplastic cells and tumor stroma. Kruskal–Wallis test followed by Dunn's multiple comparisons test was used to evaluate differences among groups. Upper panel shows F4/80+ macrophages in tumoral areas **(C)**. Bottom panel shows F4/80+ macrophages infiltrated in peritumor areas **(C)**. Macrophages quantified through expression of F4/80+ increased in 4T1 tumors on day 28, when compared to tumors on day 14 **(D)**. Results are representative of two experiments and are shown as the mean ± SEM of seven to eight animals in each group. ^*^P < 0.05, ^**^P < 0.01, respectively.

We next examined the expression of versican and TAMs infiltration evaluated in the primary tumor and metastatic lung of the 4T1 murine model of breast cancer to investigate their role during cancer progression and metastasis. Versican expression was stronger in stromal areas on the 28th day when compared with the 14th day (*P* < 0.01) (Figures 2A,B), with an increased percentage of stromal versican staining in tumors on the day 21st (*P* < 0.01) and 28th (*P* < 0.05) when compared with the 14th day. Similarly, in the neoplastic cells, stronger versican staining was found on day 28 when compared to day 14 (*P* < 0.01) (Figures 2A,B).

TAMs infiltration were also predominantly increased and distributed mainly in peritumoral areas on the 28th day when compared with the 14th day (*P* < 0.01) (Figures 2C,D). A moderate positive correlation was observed between the intensity of versican expression in neoplastic cells and TAMs density (*r* = 0.7209, *P* = 0.0036) and a moderate positive correlation with the percentage of versican expression and TAMs (*r* = 0.6299, *P* = 0.0158). No correlation was observed between stromal versican expression and TAMs (Table 1). Taken together, our data in mice and canine mammary tumors suggest an intrinsic association between stromal versican expression and TAMs infiltration with breast cancer progression and invasion.

**Table 1:** Spearman's correlation between versican expression and macrophages, cytokines, chemokines, angiogenesis, collagen deposition, and lung metastasis in the 4T1 primary tumor.

	**Versican expression in stroma areas**				**Versican expression in neoplastic cells**			
	**% Expression**		**Intensity**		**% Expression**		**Intensity**	
	**R coefficient value**	***P*-value**	**R coefficient value**	***P*-value**	**R coefficient value**	***P*-value**	**R coefficient value**	***P*-value**
**Macrophages**								
F4/80	0.3858	0.1731	0.3204	0.2444	0.6299	0.0158^*^	0.7209	0.0036^**^
**Cytokines and Chemokines**								
TNF-α	0.3314	0.2099	0.5564	0.0204^*^	0.6730	0.0043^**^	0.5649	0.0226^*^
VEGF	0.5668	0.0220^*^	0.6297	0.0067^**^	0.8489	<0.0001^***^	0.7600	0.0006^***^
CCL2	0.5878	0.0166^*^	0.6245	0.0074^**^	0.8853	0.0001^***^	0.8229	<0.0001^***^
TGF-β1	0.5715	0.0165^*^	0.6440	0.0053^**^	0.3955	0.1043	0.5232	0.0311^*^
**Angiogenesis and Collagen**								
Hemoglobin	0.4604	0.7028	0.3914	0.1202	0.7882	0.0003^***^	0.7977	0.0002^***^
N° of vessels	0.4811	0.0592	0.6440	0.0053^**^	0.9352	<0.0001^***^	0.8184	<0.0001^***^
Collagen deposition	0.4379	0.0898	0.5984	0.0087^**^	0.6857	0.0024^**^	0.6619	0.0038^**^
**Lung Metastasis**								
N° metastatic nodules	0.4135	0.1114	0.4476	0.0716	0.8495	<0.0001^***^	0.7888	0.0003^***^

*TNF-α, tumor necrosis factor-α; VEGF, vascular growth factor; CCL2, CC motif chemokine ligand 2; TGF-β1, tumor growth factor ß1*.

* *P <0.05*,

** P <0.01, and

*** *P <0.001, respectively. The bold values mean results that are statistically significant*.

### Inflammation Is Associated With Stromal Versican Expression and Peritumoral Accumulation of TGF-β1-Expressing TAMs During the 4T1 Tumor Development

**Figure 3:**
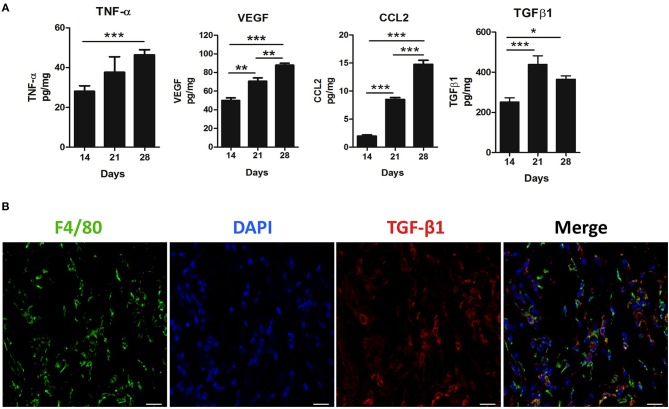
Inflammatory milieu in primary tumors during 4T1 breast carcinoma progression in mice. Levels of TNF-α, VEGF, CCL2, and TGF-β1 were measured by enzyme-linked immunosorbent (ELISA) assay on the 14th, 21st, and 28th days post tumor transplant **(A)**. One-way ANOVA test followed by Student-Newman-Keuls test was used to evaluate differences among groups. **(B)** Immunofluorescence staining of F4/80+ (green) and TGF-β1 (red) in the peritumoral area of the 4T1 primary tumor. Nuclei were counterstained with DAPI. Scale bars of 20 μm. Results are representative of two experiments and are shown as the mean ±SEM of seven to eight animals in each group. ^*^P < 0.05, ^**^P < 0.01, and ^***^P < 0.001, respectively.

We next investigated the contribution of inflammatory mediators in the 4T1 tumor microenvironment and its relationship with versican expression. A progressive increase in the pro-inflammatory cytokine TNF-α, pro-angiogenic protein VEGF, chemokine CCL2, and TGF-β1 levels were associated with the 4T1 tumor progression, with increases on days 21 and 28 when compared to day 14 (Figure 3A). A moderate to strong positive correlation was found between versican expression intensity in neoplastic cells and VEGF (*r* = 0.7600, *P* = 0.0006), CCL2 (*r* = 0.8229, *P* < 0.0001), TGF-β1 (*r* = 0.5232, *P* = 0.0311), and TNF-α (*r* = 0.5649, *P* = 0.0226) (Table 1). Versican expression percentage in neoplastic cells was positively associated with TNF-α (*r* = 0.6730, *P* = 0.0043), VEGF (*r* = 0.8489, *P* < 0.0001), and CCL2 (*r* = 0.8853, *P* = 0.0001).

A similar trend was observed between stromal versican expression intensity and TNF-α (*r* = 0.5564, *P* = 0.0204), VEGF (*r* = 0.6297, *P* = 0.0067), CCL2 (*r* = 0.6245, *P* = 0.0074), and TGF-β1 (*r* = 0.6440, *P* = 0.0053). Stromal versican expression was also positively correlated with VEGF (*r* = 0.5668, *P* = 0.0220), CCL2 (*r* = 0.5878, *P* = 0.0166), and TGF-β1 (*r* = 0.5715, *P* = 0.0165). Since versican has been previously demonstrated to be up-regulated by TGF-β in cancer scenarios (39, 40), we investigated if macrophages could be the source of secreted TGF-β1. Thus, we co-stained TGF-β1 with a murine macrophage marker, the protein F4/80 (Figure 3B), demonstrating that TGF-β1 and F4/80+ cells were predominantly localized in the peritumoral areas and, therefore, that macrophages might be an important source of TGF-β1 in the tumor microenvironment. However, other F4/80 negative cells, morphologically classified as spindle-cells resembling fibroblasts, were also found to express TGF-β1 (data not shown).

### Higher Versican Expression Is Accompanied by Angiogenesis and Tissue Remodeling During the 4T1 Tumor Development

**Figure 4:**
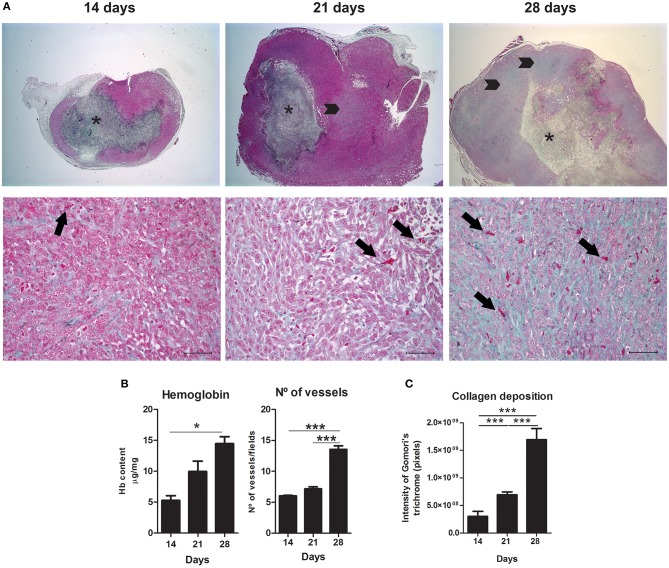
Angiogenesis and tissue remodeling during 4T1 breast carcinoma progression in mice. Slides were stained with Gomori's trichrome to evaluate angiogenesis and collagen deposition on day 14, 21, and 28 of 4T1 tumor development **(A)**. Representative images show a progressive increase in blood vessels associated with 4T1 tumor progression, quantified in **(B)** trough hemoglobin content and vessel counting, and increased in collagen deposition, quantified in **(C)**. Arrows (lower panel) represent blood vessels at different stages of tumor development. Note an increase in vessels density associated with the 4T1 tumor progression. Asterisks (upper panel) indicate central areas of necrosis with collagen deposition. Arrowheads (upper panel) represent collagen deposition in tumor areas. Note higher collagen deposition in tumoral areas at late stages. Scale bars of 50 μm. One-way ANOVA test followed by Student-Newman-Keuls test was used to evaluate differences among groups in **(B)**. Results are representative of two experiments and are shown as the mean ± SEM of seven to eight animals in each group. Kruskal–Wallis test followed by Dunn's multiple comparisons test was used to evaluate differences among groups in **(C)**. ^*^P < 0.05, ^***^P < 0.001, respectively.

The 28th day was associated with a higher amount of hemoglobin and blood vessels when compared to the 14th and 21st day (*P* < 0.05 and *P* < 0.001, respectively) (Figures 4A,B). Interestingly, 4T1 tumor progression was also associated with collagen deposition (Figures 4A,C). This tissue remodeling also appeared to be progressively higher on the 28th day of tumor progression (Figure 4C). On day 14, collagen deposition was observed most frequently in peritumoral and necrotic areas and less frequently in the versican-expressing tumor stroma. On the other hand, tumors on day 28 presented higher amount of collagen deposited mainly in the tumor stroma, in addition to peritumoral and necrotic areas (Figure 4A). Versican expression intensity and percentage in neoplastic cells was positively associated with collagen deposition (*r* = 0.6619, *P* = 0.0038 and *r* = 0.6857, *P* = 0.0024, respectively), hemoglobin content (*r* = 0.7977, *P* = 0.0002 and *r* = 0.7882, *P* = 0.0003, respectively), and blood vessels (*r* = 0.8184, *P* < 0.0001 and *r* = 0.9352, *P* < 0.0001, respectively) (Table 1). The stromal versican expression intensity was found correlated with blood vessels (*r* = 0.6440, *P* = 0.0053) and collagen deposition (*r* = 0.5984, *P* = 0.0087), while no correlation was found between stromal versican staining percentage and angiogenesis markers and collagen deposition. These results collectively indicate tissue remodeling through the development of the 4T1 tumor model associated to higher versican expression and progressive deposition of collagen.

### Pulmonary Metastasis Is Positively Correlated With Versican Expression and Macrophage Accumulation During 4T1 Mammary Cancer Progression

**Figure 5:**
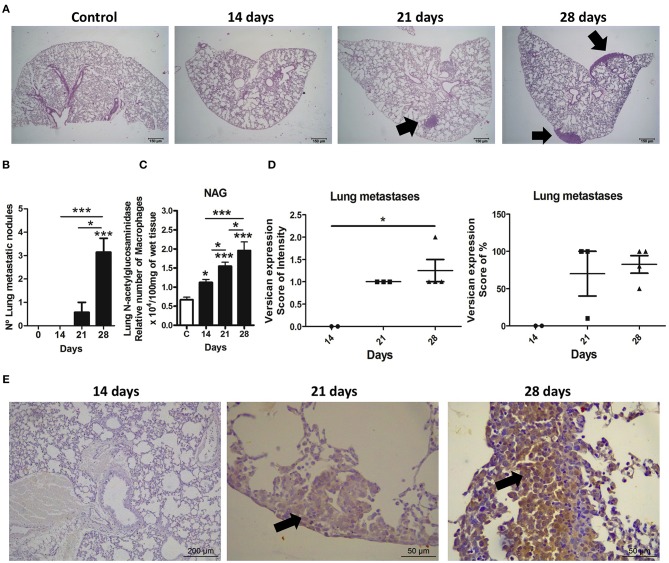
Expression and distribution of versican and metastasis in lungs during 4T1 breast carcinoma progression in mice. Lung metastases were quantified in histological slides stained with hematoxylin and eosin (H&E) at different stages of the 4T1 tumor development. Versican expression was evaluated in metastatic neoplastic cells in lung. Representative H&E images show lung sections from the control group and mice euthanatized on the 14th, 21st, and 28th day of tumor-transplant **(A)**. Arrows show neoplastic nodules present in the pleura region and lung parenchyma. Metastasis quantification shows a progressive increase in metastatic nodules **(B)**. NAG levels quantification also shows a progressive increase in macrophages in the lungs **(C)**. Versican quantification shows higher expression of this proteoglycan on metastatic nodules from animals at day 28 as compared to day 14 **(D)**. Representative immunohistochemistry images show versican expression in lung sections from the control group and mice euthanatized on the 14th, 21st, and 28th day of tumor-transplant. Arrows show versican expression in neoplastic cells present in the pleura region and lung parenchyma **(E)**. One-way ANOVA test followed by Student-Newman-Keuls test was used to evaluate differences among groups, graph **(B,C)**. Kruskal–Wallis test followed by Dunn's multiple comparisons test was used to evaluate differences among groups, graph **(D)**. Results are representative of two experiments and are shown as the mean ± SEM of seven to eight animals in each group. ^*^P < 0.05, ^***^P < 0.001, respectively.

Lung metastasis was correlated with versican expression in the 4T1 primary tumor. As expected, an increasing number of metastatic nodules following tumor development were observed (Figures 5A,B). Lung samples collected on the 28th day-post transplant showed higher numbers of metastatic nodules when compared to the day 14 (*P* < 0.001) and day 21 (*P* < 0.05) (Figures 5A,B). On the 28th day, all mice showed pulmonary metastasis, while only 37.5% (3/8) of day 21 mice presented pulmonary metastasis, and no pulmonary metastatic nodules were found in day 14 mice. Metastases were distributed throughout the lung parenchyma as nodules or small clusters of cells, mainly associated to vascular regions and pleura. Macrophage accumulation was also increased in the lung tissue in a time-dependent manner (Figure 5C). The expression of versican in neoplastic cells from primary tumors was strongly associated with the number of metastatic nodules in the lung (intensity score: *r* = 0.7888, *P* = 0.0003 and percentage score: *r* = 0.8495, *P* < 0.0001) (Table 1). Versican intensity in metastatic lung nodules was higher at day 28 when compared to day 14, while no difference was found regarding versican percentage (Figures 5D,E). Versican staining percentage in lung metastatic cells had a weak positive correlation with the number of metastatic nodules (*r* = 0.7371, *P* = 0.0370) (Table 2). No correlation was observed between NAG levels and versican expression in lung metastasis (Table 2).

**Table 2:** Spearman's correlation between versican expression and macrophages, cytokines, chemokines, and leukocytes in the 4T1 pulmonary metastases.

	**Versican expression in lung metastases**			
	**% Expression**		**Intensity**	
	**R coefficient value**	***P*-value**	**R coefficient value**	***P*-value**
**Macrophages**				
NAG	0.2394	0.5350	0.09405	0.8098
**Cytokines and Chemokines**				
CXCL1	0.1539	0.6926	0.6840	0.0422^*^
VEGF	−0.2223	0.5654	0.5643	0.1135
CCL2	0.2223	0.5654	0.7438	0.0216^*^
TGF-β1	−0.5559	0.1950	−0.3637	0.4226
**BAL**				
Total cells	0.3676	0.3304	0.3505	0.3550
Eosinophils	0.4873	0.1833	−0.4446	0.2305
Neutrophils	0.3762	0.3184	0.1624	0.6763
Macrophages	0.4959	0.1746	0.5899	0.0945
Lymphocytes	0.2052	0.5964	0.2137	0.5808
**Lung Metastasis**				
N° metastatic nodules	0.7371	0.0370^*^	0.2017	0.6319

NAG, N-acetyl-β-D-glucosaminidase; CXCL1, chemokine (C-X-C motif) ligand 1; VEGF, vascular growth factor; CCL2, CC motif chemokine ligand 2; TGF-β1, tumor growth factor ß1; BAL, bronchoalveolar lavage.

* P <0.05, ^***^P <0.001, respectively. The bold values mean results that are statistically significant.

### High Versican Expression in Lung Parenchyma Was Associated With Progressive Leukocyte Influx and Pulmonary Dysfunction During 4T1 Mammary Cancer Metastasis

**Figure 6:**
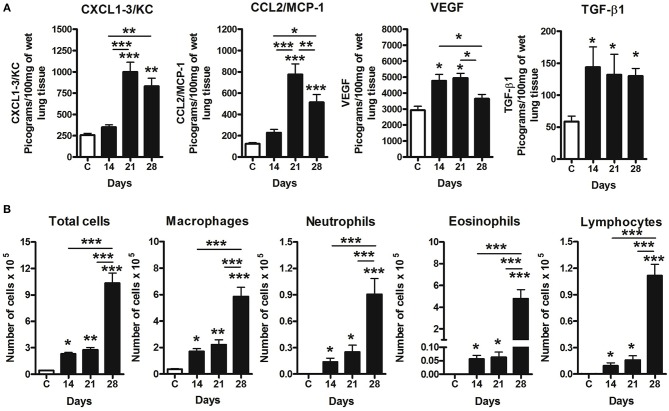
Pulmonary inflammation related to 4T1 mammary carcinoma metastasis progression in mice. Inflammation was evaluated in BAL and differential leukocyte counts and ELISA assays in the lungs samples. Levels of CXCL1, CCL2, VEGF, and TGF-β1 were measured by ELISA assay on the 14th, 21st, and 28th days post tumor transplant **(A)**. BAL counts shows a progressive leukocyte infiltration following metastatic development of the 4T1 tumor, with a predominance of macrophages in BAL in late time-points, when compared to Control group **(B)**. One-way ANOVA test followed by Student-Newman-Keuls test was used to evaluate differences among groups. Results are representative of two experiments and are shown as the mean ± SEM of seven to eight animals in each group. ^*^P < 0.05, ^**^P < 0.01, and ^***^P < 0.001, respectively.

Cytokines and chemokines were measured in lung tissue (Figure 6A). CCL2 and CXCL1 peaks were detectable from day 21, dropping slightly on day 28. VEGF was increased on days 14 and 21, decreasing on day 2. TGF-β1 was found increased in all tumor-bearing mice when compared to the control animals. Versican staining intensity was correlated with CXCL1 (*r* = 0.6840, *P* = 0.0422) and CCL2 (*r* = 0.7438, *P* = 0.0216) levels in the lung parenchyma (Table 2). No association was observed between versican expression and VEGF or TGF-β1 (Table 2).

Airway leukocyte influx was evaluated in BAL fluid, which revealed a progressive increase of leukocytes (Figure 6B) associated to tumor development and metastasis. All inflammatory cell types were increased in BAL at days 14, 21, and 28 when compared to the control. Day 14 was marked by a predominance of macrophage, and neutrophil, eosinophil and lymphocyte influx on days 21 and 28 (Figure 6B). Moreover, total leukocytes peaked on day 28 when compared to day 14 and 21, with a predominance of macrophages and eosinophils during late stages of metastatic progression (Figure 6B). No association was observed between versican expression in metastatic nodules and leukocytes presented in BAL.

**Figure 7:**
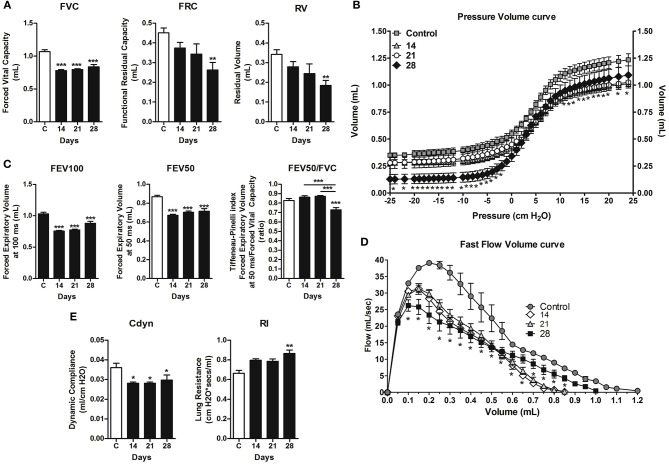
Assessment of pulmonary mechanic functions at different stages of lung metastasis during 4T1 breast carcinoma metastasis progression in mice. Invasive spirometry was performed to investigate functional modifications in pulmonary mechanics. The parameters assessed were: Lung volumes **(A)**, depicted by Forced Vital Capacity, Functional Residual Capacity, and Residual Volume; Pressure × Volume Curve **(B)**; Airway flow **(C)** by Forced Expiratory Volume, Forced Expiratory Volume at 50 ms, and Tiffeneau-Pinelli index; Fast Flow Volume Curve **(D)**; Lung elasticity **(E)** by Dynamic Compliance Forced and Lung Resistance. Kruskal–Wallis test followed by Dunn's multiple comparisons test was used to evaluate differences among groups. Results are shown as the mean ± SEM. ^*^P < 0.05; ^**^P < 0.01; ^***^P < 0.001, respectively.

A forced spirometry technique was used to evaluate the physiological changes in lung function caused by the metastatic development and versican accumulation in lung parenchyma during 4T1 breast cancer metastasis. High versican expression in parallel with metastatic progression leads to a progressive decline in pulmonary function, mainly highlighted on day 28, evidenced by volume loss, such as reduction in FVC, FRC, RV (Figure 7A), and the Pressure × Volume curve (Figure 7B). In addition, the mice presented a time-dependent decline in respiratory airway flow, with a decrease in FEV50 and in FEV100 (Figure 7C), confirmed by the Flow × Volume curve, when compared to the control animals (Figure 7D). Moreover, the day 28 animals presented a greater restrictive behavior of the respiratory airway flow, evidenced by the Tiffeneau-Pinelli index (Figure 7C). Regarding pulmonary elasticity, through analysis of lung compliance (Cdyn) and resistance (Rl), animals transplanted with the 4T1 tumors exhibited a reduction in dynamic compliance, with a significant increase in resistance on day 28 when compared to control animals (Figure 7E). Collectively, our data suggests that the lost pulmonary function may is a result from the accumulation of pulmonary metastatic nodes on day 28.

## Discussion

Versican expression has been linked with poor prognosis and relapse-free survival in cancer patients (15, 41, 42). Interaction among versican, ECM components, and neoplastic cells are recognized as regulators of proliferation and metastatic potential (16, 43, 44). Indeed, immune cells, mostly macrophages, have an important role in ECM remodeling, including regulation of versican levels and, consequently, tumor progression (3, 44). A previous study described the interaction of versican with TAMs, promoting their activation, migration, and production of growth factors (20). However, in the context of breast cancer, the most common cancer in women worldwide and cancer-related deaths in women (7), the role of versican remains unexplored. Here we highlighted the correlation between versican expression, TAMs accumulation and tumor progression using two models of mammary carcinomas: spontaneous mammary carcinoma in dogs and mammary carcinoma implanted in mice. Naturally occurring cancers in canines and humans share many features, including tumor phenotype, genetic markers and molecular targets (45). Thus, studying the cancer development in canines and rodents may provide a valuable perspective, potentially useful for diagnosis and therapeutics in humans.

The tumor microenvironment is an important participant of tumor progression and metastasis (3). TAMs are important in the context of tumor biology, involved in both invasiveness and metastatization (21–23), with M2-polarized macrophages characterized as promoters of angiogenesis, epithelial-mesenchymal transition and tissue remodeling in cancer manifestation (27). We showed that TAMs were predominately localized in the peripheral areas of primary tumors, with their increase associated to stromal versican expression, as observed by Asano et al. (46). M2-polarized macrophages have been showed to produce large quantities of soluble mediators in the tumor microenvironment such as CCL2, VEGF, and TGF-β1 that interact with neoplastic cells promoting their proliferation and invasion (47–51). However, we found increased levels of CCL2, VEGF, and TGF-β1 also positively correlating with F4/80+ macrophages, high vascularization and collagen deposition during tumor progression in the 4T1 mouse model. These macrophages are the source of TGF-β1, as confirmed by microscopy. TAMs can also induce tumor angiogenesis, and have been involved in collagen deposition and linearization of the tumor stroma in more aggressive human breast cancer subtypes (8). Accordingly, we revealed that the 4T1 development evolved in a TAM-derived inflamed milieu and neovascularized tumor tissue, with increased collagen deposition in the tumor microenvironment. Collectively, these findings indicate that the 4T1 tumor progression is related with peritumoral TAM infiltration and may promote the gradual replacement of extracellular matrix and angiogenesis through its cytokines production.

Versican, an extracellular matrix proteoglycan, is also involved in TAM activation through TLRs, inducing the production of pro-inflammatory and pro-tumoral cytokines and chemokines, in addition to versican production (17–20, 50). In fact, these interactions between tissue microenvironment and parenchymal cells are detrimental for the maintenance of cancer progression and invasiveness. In breast cancer, the interaction between stromal versican deposition and neoplastic cells was previously shown as an important step in tumorigenesis (46). However, the authors described low proteoglycan expression in the cells, possibly justified by the secretion of soluble mediators by other cells, leading to an increase in the deposition of versican by fibroblasts during neoplastic extracellular matrix remodeling. Besides fibroblasts, macrophages have also been demonstrated to produce versican in different murine tumor models (46).

Breast cancer metastasizes through lymphatic and blood vessels mainly to lymph nodes, lungs, bones, liver, and brain (52, 53). The onset of metastasis in the 4T1 tumor often occurs after the second week post-transplant (54). In the present study, versican expression was observed in neoplastic cells and peripheral areas in all studied time-points. The metastatic progression of the 4T1 tumor was accompanied by the up-regulation of versican expression in neoplastic cells of the primary tumor, which was correlated with the number of pulmonary metastatic nodules. In fact, overexpression of versican has been showed to increase growth, proliferation and metastatic potential of a breast cancer murine model (43), while the versican silencing exerted an inhibitory effect on the proliferative and migratory ability of melanoma cells (55). Thus, since versican was differentially expressed in peripheral and tumor areas, we suggest a pro-tumoral role of versican, promoting tumor progression and metastasis of the 4T1 tumor model.

Versican has been shown to interact with myeloid and lymphoid cells in the tumor microenvironment, promoting their adhesion and production of inflammatory cytokines that may trigger the tumor invasion (11, 16, 19, 56, 57). Thus, the proteoglycan versican may interact with leukocytes and neoplastic cells to establish a microenvironment that favors tumor expansion in the context of the 4T1 model. Increased cytokines and chemokines, such as TGF-β1, CCL2, and VEGF, have been shown to predict metastatic disease (50, 51, 53). Furthermore, TGF-β1 has been shown to up-regulate the production of versican in cancer cells and the tumor stroma, resulting in invasiveness and metastasis (39, 40, 49). We found a marked production of CCL2 and CXCL1 in the lung, in parallel with macrophage influx, increased metastatic disease and high versican expression in the lung tissue. These chemokines are related to leukocyte recruitment and angiogenesis, which could contribute toward a pro-metastatic pulmonary microenvironment, directly stimulate the tumor progression and metastasis.

Lung metastasis may affect pulmonary microenvironment and function (10). The progression of the 4T1 breast cancer model resulted in an increased leukocyte influx in the airways, mostly composed by macrophages, also associated to a higher number of metastatic nodules and infiltrating TAMs. Pulmonary mechanical functions were altered during tumor progression. This alteration was evidenced even on day 14, when metastatic nodules were absent, and progressed to more pronounced lung dysfunction on day 28. Our data corroborates that pulmonary versican expression is associated to metastatic nodules, inflammation and alveolar edema, which impacts lung dysfunction, preceding mortality. Moreover, the number of metastatic nodes in the lung leads to a greater restrictive behavior of the respiratory airway flow in mice. Finally, future studies using cytokines- and versican-knockdown mice and macrophage-depleted mice could better explore the relationship between TAMs and versican in the metastatic development of 4T1 breast cancer mice model.

In conclusion, stromal versican expression was found in primary canine and murine breast cancer models. Increased versican expression correlates with elevated TAMs infiltration in the primary tumor preceding the metastatic progression in mice. Versican expression also correlates with increase metastasis and pulmonary dysfunction, suggesting the involvement of TAMs in regulating important steps during murine 4T1 breast cancer model development through versican signaling. Therefore, versican and TAMs might represent an attractive target for breast cancer therapy.

## Ethics Statement

Canine mammary tumors samples: All procedures were performed under the guidelines and with the approval of the Ethics Committee in Animal Experimentation (CETEA/UFMG), protocol 219/2009 and 81/2013). Murine 4T1 breast cancer model: All research was conducted under a protocol approved (number 262/2012) by an Ethics Committee on Animal Use (CEUA) from Federal University of Minas Gerais, Brazil.

## Author Contributions

GC, RR, and DdR designed the research. DdR performed *in vivo* experiments, histology image analysis, and data analysis. KD performed immunohistochemistry, score, and analyses of versican and macrophages in canine samples. EV and GP maintained the BALB/c mice used in the experiments and performed mouse necropsies. LK, MM, and PC performed BAL, cytokines, and chemokines dosages. RR executed and analyzed forced spirometry data. MR and DG were responsible by confocal image. DdR, KD, CdC, EF, RR, and GC drafted the work. GC and RR supervised the study and final approval of the version to be published.

### Conflict of Interest Statement

The authors declare that the research was conducted in the absence of any commercial or financial relationships that could be construed as a potential conflict of interest.
